# Simple Synthesis of Modafinil Derivatives and Their Anti-inflammatory Activity

**DOI:** 10.3390/molecules170910446

**Published:** 2012-09-03

**Authors:** Jae-Chul Jung, Yeonju Lee, Jee-Young Son, Eunyoung Lim, Mankil Jung, Seikwan Oh

**Affiliations:** 1 Department of Neuroscience and Tissue Injury Defense Research Center, School of Medicine, Ewha Womans University, Seoul 158-710, Korea; 2 Department of Chemistry, Yonsei University, Seoul 120-749, Korea

**Keywords:** modafinil, anti-inflammation, nitric oxide, *S*-alkylation, coupling reaction

## Abstract

Simple synthesis of modafinil derivatives and their biological activity are described. The key synthetic strategies involve substitution and coupling reactions. We determined the anti-inflammatory effects of modafinil derivatives in cultured BV2 cells by measuring the inhibition of nitrite production and expression of iNOS and COX-2 after LPS stimulation. It was found that for sulfide analogues introduction of aliphatic groups on the amide part (compounds **11a**–**d**) resulted in lower anti-inflammatory activity compared with cyclic or aromatic moieties (compounds **11e**–**k**). However, for the sulfoxide analogues, introduction of aliphatic moieties (compounds **12a**–**d**) showed higher anti-inflammatory activity than cyclic or aromatic fragments (compounds **12e**–**k**) in BV-2 microglia cells.

## 1. Introduction

**Figure 1:**
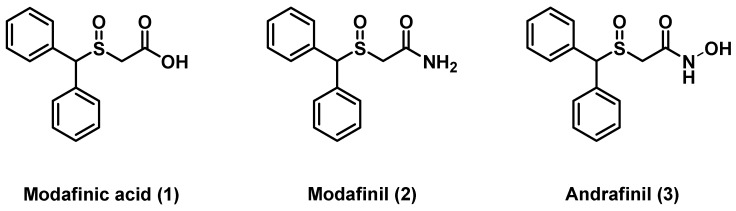
Structures of modafinic acid (**1**), modafinil (**2**) and andrafinil (**3**).

Modafinil in racemic form is proving clinically useful in the treatment of narcolepsy, a neurological disorder marked by uncontrollable attacks of daytime sleepiness [1]. Since the discovery of (±)-2-(diphenylmethylsulfinyl)acetamide by Laboratoire L. Lafon, modafinil and its derivatives (Figure 1) have stimulated significant interest due to their potential biological activities [2]. It is experimentally used in the treatment of Alzheimer's disease, myotonic dystrophy, multiple sclerosis-induced fatigue, jet-lag, and cognitive impairment in schizophrenia [3,4,5].

Since modafinil has been used as a psychostimulant for the treatment of narcolepsy, most research on the action mechanism of modafinil has focused on its monoaminergic effects, showing that modafinil stimulates the dopamine, serotonin, and norepinephrine pathways in the brain. In addition, modafinil is known to inhibit hepatic cytochrome P450 activities and has a neuroprotective function [6,7,8]. It might also be useful to treat attention deficit hyperactivity disorder (ADHD), and cancer-related fatigue [9,10]. Recently, other therapeutic applications of modafinil include Parkinson’s disease, and cocaine dependence and withdrawal [11,12,13]. A link between systemic inflammation and dementia was first hypothesized after discovery of up-regulated inflammatory processes localized to Alzheimer’s disease pathology in post-mortem brain specimens [14]. Given the role of glia in mediating neurodegeneration, a great deal of effort has been made to develop novel treatment for Parkinson’s disease by targeting glia and associated inflammatory factors [15,16]. These results led us to investigate the pharmacological activity of modafinil derivatives on the inflammation.

Recent reports described a simple synthesis of racemic or single isomer modafinil and its derivatives, including their screening assay for their biological properties [3,4]. The Prisinzano group reported asymmetric synthesis and elucidation of the absolute configuration of the enantiomers of modafinil [17]. The Olivo group has developed a preparation of (*S*)-modafinil in 68% yield via microbial (*Bacillus subtilis*) oxidation and subsequent amidation of benzhydrylsulfanyl acetic acid [18]. The Coquerel group demonstrated an enantioselective synthesis of modafinil and its derivatives using an enantioselective oxidation with stoichiometric chiral oxaziridine in 66% yield [19]. The Woster group has described polyaminohydroxamides and polyaminobenzamides as isoform selective histone deacetylase (HDAC) inhibitors [20].

In the context of our medicinal research program dealing with the synthesis of biologically active modafinil derivatives, we wish to report a simple synthesis of modafinil analogues from benzophenone through intramolecular ring cyclization, *O*-alkylation, and coupling reaction. Biological activities of these modafinil analogues for suppression of LPS-induced NO generation and the expression of inflammation-related enzymes were measured in BV2 cells. These results suggest that modafinil derivatives can also be developed as potential anti-inflammatory agents.

**Scheme 1:**
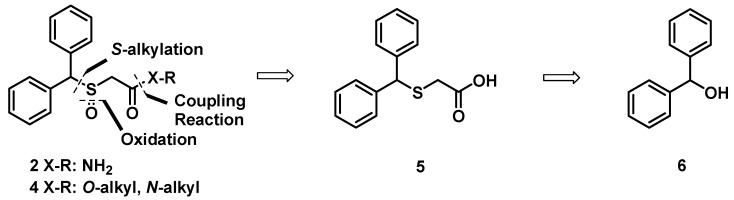
Retrosynthetic analysis of the modafinil (**2**) and its derivatives **4**.

Scheme 1 reveals our overall retro-synthetic approach for these target molecules. The benzothiol group skeleton is an essential structural feature for the modafinil moieties of narcolepsy agents. They have traditionally been constructed by substitution and hydrolysis in acidic media. The key intermediate **5** was obtained by *S*-alkylation of benzhydrol (**6**), which could be oxidized to generate modafinil (**2**). Acids **7** and **1** were treated with azide and then oxidized to generate modafinil derivatives.

## 2. Results and Discussion

### 2.1. Chemistry

**Scheme 2:**
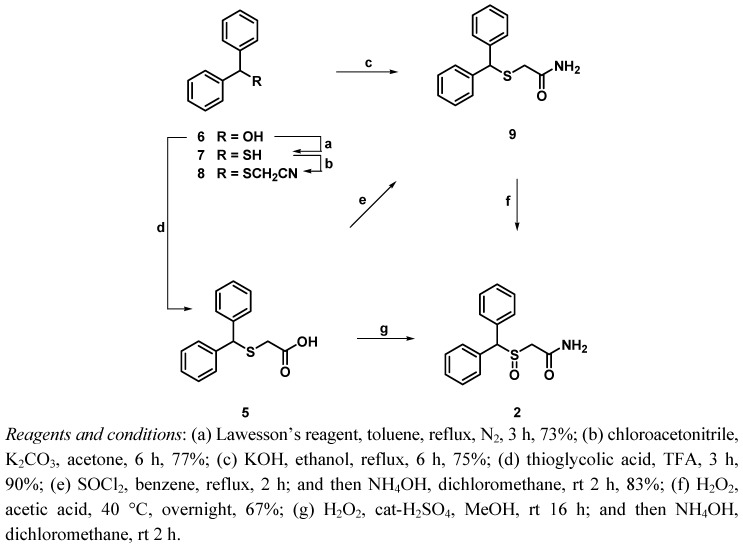
Synthesis of modafinil.

To generate key fragment **5**, which is well known as a pharmacophore for psycostimulant agents, commercially available benzhydrol (**6**) was treated with Lawesson’s reagent in toluene under nitrogen atmosphere to give **7** in 73% yield [21]. Substitution of thiol **7** was performed with chloroacetonitrile in the presence of potassium carbonate in acetone to give nitrile **8** in 77% yield. Compound **7** was treated with potassium hydroxide in ethanol via oxidative hydration to give acetamide **9** in 75% yield. Subsequent oxidation of acetamide **9** was accomplished with 30%-H_2_O_2_ in acetic acid in order to afford modafinil (**2**) in 67% yield (Scheme 2).

On the other hand, direct alkylation of benzhydrol (**6**) with thioglycolic acid in TFA gave acid **5** in 90% yield. In an attempt to prepare modafinil (**2**) from benzhydrylsulfanyl acetic acid **5** it was treated with H_2_O_2_ in acidic media and then treated with NH_4_OH in dichloromethane, but this reaction was unsatisfactory, and for the most part the starting material was recovered. Acid **5** was however smoothly transformed into acetamide **9** in 83% yield through treatment with thionyl chloride and ammonium hydroxide in a two step process [22]. Oxidation of compound **9** was accomplished with H_2_O_2_ in acetic acid at 40 °C to generate modafinil (**2**) in 67% yield (Scheme 2).

**Scheme 3:**
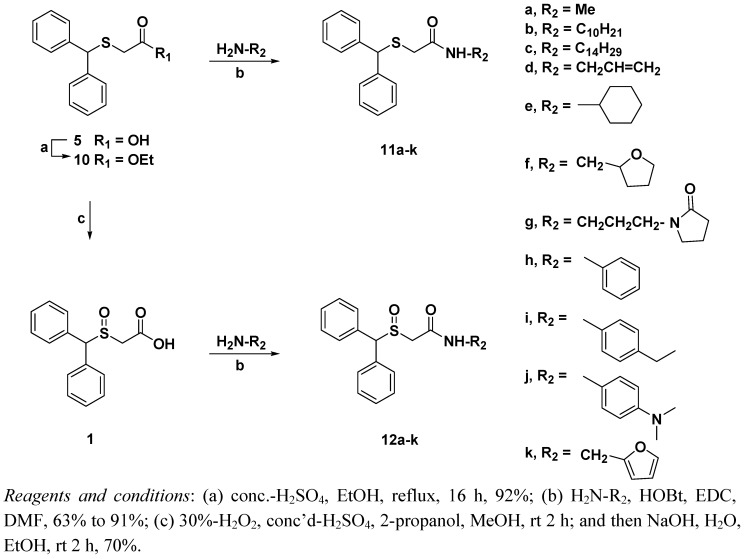
Synthesis of modafinil derivatives.

Esterification of acid **5** was performed by concentrated H_2_SO_4_ in ethanol to give ester **10** in 92% yield. Compound **5** was also treated with 30%-H_2_O_2_ in the presence of conc. H_2_SO_4_ in methanol to afford the sulfonate, which was subsequently hydrolyzed using aqueous sodium hydroxide in ethanol to generate acid **1** in 70% yield. Acids **5** and **1** were finally treated with various amines (alkyl amines: methylamine, allylamine, *n*-decylamine, *t*-decylamine, 1-(3-aminopropyl)pyrrolidinone, cyclohexylamine; heterocyclic amines: tetrahydrofurfurylamine, and furfurylamine, and anilines: aniline, 4-ethylaniline, *N*,*N*-dimethyl-*p*-phenylenediamine) in the presence of 1-hydroxybenzotriazole (HOBt), and ethyl(dimethylaminopropyl)-carbodiimide (EDC) in DMF to give modafinil derivatives **11a–k** and **12a–k**, respectively (Scheme 3).

### 2.2. Biology

**Figure 2:**
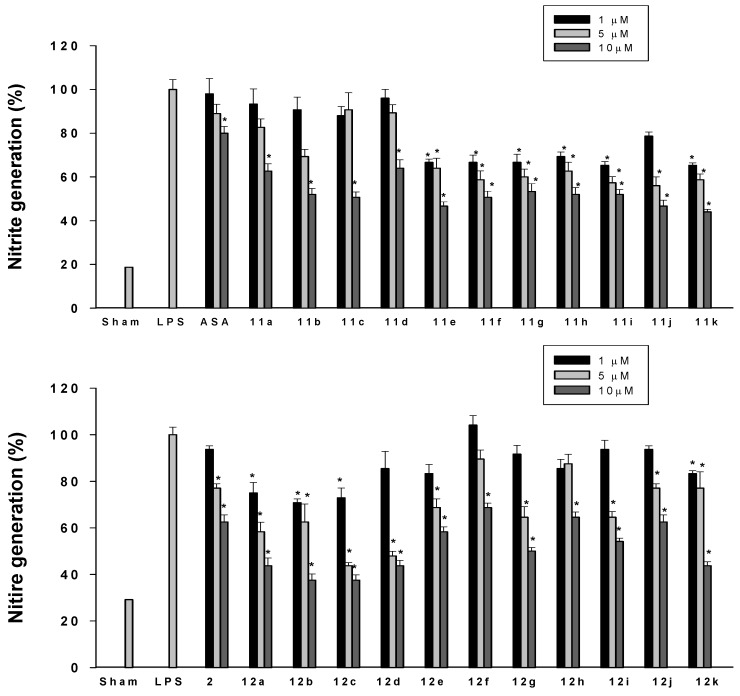
Effect of modafinil derivatives **11a–k**, **12a–k** and aspirin (ASA) on nitrite production in LPS-stimulated BV-2 microglia cells. Cells were treated with 100 ng/mL LPS, and then various concentrations of test compounds (1 µM, 5 µM, and 10 µM) were added for 24 h at 37 °C. Values indicate nitrite production from culture supernatants of LPS-treated cells with or without compounds. Data represent the mean ± standard deviation of three observations. * *p* < 0.05 in comparison with LPS treated group.

Nitrite was used as a measure of NO production as a marker of inflammation induction. The *in vitro* suppression of LPS-induced NO generation of the prepared modafinil derivatives was evaluated by the published test method [23] and the results are summarized in Figure 2. Chen *et al.* have reported that aspirin and indomethacin both decrease NOS activity and nitrite/nitrate formation in platelets [24]. Aspirin was used as reference anti-inflammatory agent in this experiment. Most of the modafinil derivatives inhibited nitrite accumulation in LPS-stimulated microglia BV-2 cells to some extent, with the aliphatic derivatives **12a–d** exhibiting the greatest inhibitory activity for LPS-induced NO generation. Interestingly, compounds **11a–d** (with aliphatic groups and without sulfoxide) showed very low activity in inhibition of NO production at 1–5 µM concentrations in LPS-stimulated BV-2 cells (Figure 2).

### 2.3. Reduction of the Level of Pro-Inflammatory Enzyme Expression

**Figure 3:**
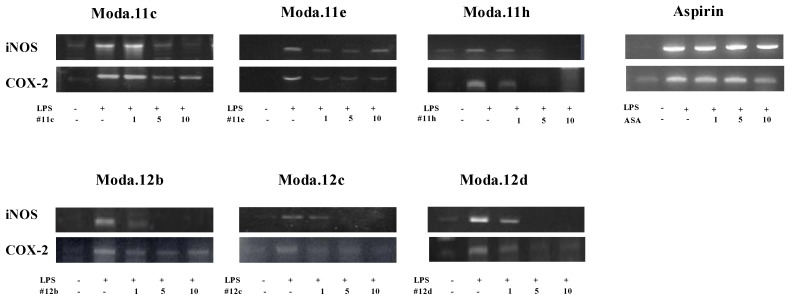
Effects of modafinil derivatives and aspirin (ASA) on inflammation-related enzyme mRNA expression in LPS-treated BV2 cells. Cells were treated by 100ng/ml LPS with or without modafinil derivatives or aspirin (1 µM, 5 µM, and 10 µM) for 24 h. Expression of iNOS and COX-2 was measured by PCR analysis.

Modafinil derivatives exerted an anti-inflammatory effect on LPS-induced responses accompanied by the expression of pro-inflammatory enzymes in cells. BV2 cells were collected after activation by LPS (100 ng/mL) with or without modafinil derivatives (1 µM, 5 µM, and 10 µM) for 24 h. The mRNA expression levels of the iNOS and COX-2 were reduced by treatment with modafinil derivatives **11c, 11e, 11h** and **12b–d** (Figure 3). Interestingly, modafinil derivatives **12b–d** highly suppressed the LPS-induced iNOS expression. These results indicated that modafinil derivatives have anti-inflammatory effects on the expression of LPS-induced pro-inflammatory enzymes in cultured cells.

## 3. Experimental

### 3.1. General Procedures

Reactions requiring anhydrous conditions were performed with the usual precautions for rigorous exclusion of air and moisture. Tetrahydrofuran was distilled from sodium benzophenone ketyl prior to use. Thin layer chromatography (TLC) was performed on precoated silica gel G and GP uniplate from Analtech and visualized with 254-nm UV light. Flash chromatography was carried out on silica gel 60 [Scientific Adsorbents Incorporated (SAI), particle size 32–63 µm, pore size 60 Å]. ^1^H-NMR and^ 13^C-NMR spectra were recorded on a Bruker DPX 250 instrument at 250 MHz and 63 MHz, respectively. The chemical shifts are reported in parts per million (ppm) downfield from tetramethylsilane, and *J* values are in Hz. Infrared (IR) spectra were obtained on an ATI Mattson FT/IR spectrometer. Mass spectra were recorded with a Waters Micromass ZQ LC-Mass system and high resolution mass spectra (HRMS) were measured with a Bruker BioApex FTMS system by direct injection using an electrospray interface (ESI). When necessary, chemicals were purified according to the reported procedures [25].

### 3.2. General Procedure for the Preparation of Compounds ***11a–k*** and ***12a–k*** via Condensation of Acids ***1, 5*** and Amines

To a stirred solution of acids (**1** or **5**; 0.077 mmol) and HOBt (20.8 mg, 0.154 mmol) EDC (29.5 mg, 0.154 mmol) in DMF (4 mL) was added at room temperature and the mixture was stirred for 30 min at same temperature. The appropriate amine (0.077 mmol) was added by syringe to the reaction mixture which was then stirred at room temperature for 1 h. The reaction mixture was diluted with ethyl acetate (10 mL) and washed with brine (10 mL). The organic layer was separated, dried over anhydrous MgSO_4_, filtered and concentrated under reduced pressure. The product was purified by flash column chromatography on silica gel (*n*-hexane/EtOAc = 2:1, v/v) to give **11a–k** and **12a–k**.

*2-Benzyhydrylsulfanyl-N-methylacetamide* (**11a**). White solid. mp 99.0 °C; R*_f_* = 0.27 (ethyl acetate/*n*-hexane = 1:1, v/v); IR _max_ (CHCl_3_, KBr): 2922, 2850, 1736, 1493, 1449, 1269, 1143, 1125, 1050, 1030, 970, 750 cm^−1^; ^1^H-NMR (CDCl_3_): δ 7.42–7.21 (m, 10H), 6.60 (s, 1H), 5.12 (s, 1H), 3.11 (s, 2H), 2.75–2.73 (d, *J* = 4.8 Hz, 3H); ^13^C-NMR (CDCl_3_): δ 169.1, 140.6, 128.9, 128.4, 127.8, 55.3, 36.2, 26.7; LC-MS (ESI+) *m/z* 294 - [M+Na].

*2-Benzyhydrylsulfanyl-N-decylacetamide* (**11b**). White liquid. R*_f_* = 0.4 (ethyl acetate/*n*-hexane = 1:2, v/v); IR _max_ (CHCl_3_, KBr): 3466, 3300, 3005, 2926, 2129, 1742, 1600, 1496, 1451, 1384, 1280, 1158, 1117, 1055, 995, 754, cm^−1^; ^1^H-NMR (CDCl_3_): δ 7.41–7.21 (m, 10H), 6.65 (s, 1H), 5.11 (s, 1H), 3.24–3.16 (q, *J* = 4.2 Hz, 2H), 3.11 (s, 2H), 1.27 (brs, 16H), 0.91–0.86 (t, *J* = 3.8 Hz, 3H); ^13^C-NMR (CDCl_3_): δ 168.3, 140.6, 129.0, 128.4, 127.8, 55.3, 40.0, 36.4, 32.1, 31.1, 29.5, 27.2, 22.9, 14.3; LC-MS (ESI+) *m/z* 420 - [M+Na].

*2-Benzyhydrylsulfanyl-N-tetradecylacetamide* (**11c**). White solid. mp 52.2 °C; R*_f_* = 0.43 (ethyl acetate/*n*-hexane = 1:2, v/v); IR _max_ (CHCl_3_, KBr): 3302, 2963, 2925, 1658, 1600, 1516, 1450, 1412, 1324, 1248, 1077, 1030, 833, 750 cm^−1^; ^1^H-NMR (CDCl_3_): δ 7.42–7.21 (m, 10H), 6.64 (s, 1H), 5.11 (s, 1H), 3.24–3.16 (q, *J* = 4.5 Hz, 2H), 3.11 (s, 2H), 1.26 (brs, 24H), 0.90–0.85 (t, *J* = 4.0 Hz, 3H); ^13^C-NMR (CDCl_3_): δ 167.7, 140.6, 129.0, 128.4, 127.8, 55.3, 40.0, 36.4, 32.1, 31.1, 29.8, 29.6, 27.2, 22.9, 14.1; LC-MS (ESI+) *m/z* 476 - [M+Na].

*N-Allyl-2-benzyhydrylsulfanylacetamide* (**11d**). Light yellow liquid. R*_f_* = 0.47 (ethyl acetate/*n*-hexane = 1:1, v/v); IR _max_ (CHCl_3_, KBr): 3297, 3026, 2924, 1650, 1541, 1493, 1450, 1410, 1318, 1162, 1077, 1030, 750 cm^−1^; ^1^H-NMR (CDCl_3_): δ 7.42–7.21 (m, 10H), 6.71 (s, 1H), 5.89–5.73 (m, 1H), 5.23–5.14 (m, 3H), 3.85–3.80 (t, *J* = 5.1 Hz, 2H), 3.13 (s, 2H); ^13^C-NMR (CDCl_3_): δ 168.3, 140.5, 134.0, 128.4, 127.8, 127.1, 116.8, 42.3, 36.3, 31.1; LC-MS (ESI+) *m/z* 320 - [M+Na].

*2-Benzyhydrylsulfanyl-N-cyclohexylacetamide* (**11e**). White solid. mp 120.9 °C; R*_f_* = 0.67 (ethyl acetate/*n*-hexane = 1:1, v/v); IR_max_ (CHCl_3_, KBr): 3291, 3061, 3027, 2928, 2853, 1643, 1548, 1494, 1450, 1325, 1246, 1153, 1077, 1030, 986, 891, 749 cm^−1^; ^1^H-NMR (CDCl_3_): δ 7.69–7.24 (m, 10H), 6.59 (s, 1H), 5.11 (s, 1H), 3.77–3.74 (m, 1H), 3.07 (s, 2H), 1.74–1.67 (q, *J* = 6.5 Hz, 4H), 1.35–1.23 (m, 6H); ^13^C-NMR (CDCl_3_): δ 167.2, 140.4, 128.9, 128.4, 127.7, 55.0, 48.5, 36.3, 33.1, 29.8, 25.6; LC-MS (ESI+) *m/z* 362 - [M+Na].

*2-Benzyhydrylsulfanyl-N-(tetrahydrofuran-2-ylmethyl)acetamide* (**11f**). White solid. mp 117.8 °C; R*_f_* = 0.3 (ethyl acetate/*n*-hexane = 1:1, v/v); IR _max_ (CHCl_3_, KBr): 3304, 3060, 3027, 2926, 2870, 1651, 1531, 1494, 1450, 1384, 1304, 1078, 1030, 922, 750 cm^−1^; ^1^H-NMR (CDCl_3_): δ 7.43–7.20 (m, 10H), 6.99 (s, 1H), 5.20 (s, 1H), 3.99–3.75 (m, 3H), 3.58–3.52 (m, 1H), 3.19–3.09 (m, 3H), 2.02–1.87 (m, 4H); ^13^C-NMR (CDCl_3_): δ 168.5, 140.3, 128.8, 128.4, 127.6, 68.2, 54.8, 43.5, 36.1, 29.8, 25.9; LC-MS (ESI+) *m/z* 364 - [M+Na].

*2-Benzyhydrylsulfanyl-N-[3-(2-oxo-pyrrolidin-1-yl)-propyl]acetamide* (**11g**). Light yellow liquid. R*_f_* = 0.3 (dichloromethame/methanol = 10:1, v/v); IR _max_ (CHCl_3_, KBr): 3293, 3060, 3027, 2928, 2870, 1738, 1660, 1539, 1494, 1450, 1383, 1292, 1248, 1078, 1030, 751 cm^−1^; ^1^H-NMR (CDCl_3_): δ 7.46–7.21 (m, 11H), 5.30 (s, 1H), 3.39–3.08 (m, 8H), 2.43–2.36 (t, *J* = 5.0 Hz, 2H), 2.08–1.96 (m, 2H), 1.71–1.61 (m, 2H); ^13^C-NMR (CDCl_3_): δ 175.8, 169.0, 140.6, 128.7, 128.5, 127.5, 54.7, 47.3, 40.8, 36.4, 36.1, 30.9, 26.8, 18.0; LC-MS (ESI+) *m/z* 405 - [M+Na].

*2-Benzyhydrylsulfanyl-N-phenylacetamide* (**11h**). White solid. mp 98.9 °C; R*_f_* = 0.43 (ethyl acetate/*n*-hexane = 1:2, v/v); IR _max_ (CHCl_3_, KBr): 3301, 3060, 3019, 2913, 1659, 1601, 1540, 1495, 1442, 1319, 1242, 1074, 1029, 747 cm^−1^; ^1^H-NMR (CDCl_3_): δ 8.45 (s, 1H), 7.49–7.08 (m, 15H), 5.19 (s, 1H), 3.23 (s, 2H); ^13^C-NMR (CDCl_3_): δ 166.7, 140.3, 137.7, 129.2, 129.0, 128.5, 127.9, 124.8, 119.9, 54.3, 37.2; LC-MS (ESI+) *m/z* 356 - [M+Na].

*2-Benzyhydrylsulfanyl-N-(4-ethylphenyl)acetamide* (**11i**). White solid. mp 90.4 °C; R*_f_* = 0.5 (ethyl acetate/*n*-hexane = 1:2, v/v); IR _max_ (CHCl_3_, KBr): 3440, 2922, 2850, 1738, 1495, 1450, 1384, 1282, 1153, 1117, 1053, 1032, 967, 752, 704 cm^−1^; ^1^H-NMR (CDCl_3_): δ 7.42–7.26 (m, 12H), 7.21 (s, 1H), 7.17–7.14 (d, *J* = 7.1 Hz, 2H), 5.18 (s, 1H), 3.23 (s, 2H), 2.66–2.57 (q, *J* = 4.9 Hz, 2H), 1.25–1.12 (t, *J* = 4.2 Hz, 3H); ^13^C-NMR (CDCl_3_): δ 166.4, 140.8, 140.2, 135.2, 128.9, 128.4, 128.3, 127.8, 120.0, 55.2, 37.1, 28.4, 15.8; LC-MS (ESI+) *m/z* 384 - [M+Na].

*2-Benzyhydrylsulfanyl-N-(4-dimethylaminophenyl)acetamide* (**11j**). Light yellow solid. mp 131.8 °C; R*_f_* = 0.53 (ethyl acetate/*n*-hexane = 1:1, v/v); IR _max_ (CHCl_3_, KBr): 3304, 2918, 1651, 1600, 1519, 1450, 1384, 1324, 1248, 1125, 1029, 947, 816, 749 cm^−1^; ^1^H-NMR (CDCl_3_): δ 8.29 (s, 1H), 7.44–7.24 (m, 12H), 6.73–6.70 (d, *J* = 7.5 Hz, 2H), 5.12 (s, 1H), 3.23 (s, 2H), 2.93 (s, 6H); ^13^C-NMR (CDCl_3_): δ 166.1, 140.4, 128.4, 127.8, 127.2, 121.8, 113.2, 41.1, 37.0, 29.8; LC-MS (ESI+) *m/z* 399- [M+Na].

*2-Benzyhydrylsulfanyl-N-(furan-2-ylmethyl)acetamide* (**11k**). Light yellow solid. mp 89.6 °C; R*_f_* = 0.27 (ethyl acetate/*n*-hexane = 1:2, v/v); IR _max_ (CHCl_3_, KBr): 3291, 3059, 3026, 2923, 1648, 1559, 1541, 1522, 1508, 1491, 1449, 1419, 1385, 1313, 1195, 1147, 1078, 1016, 748 cm^−1^; ^1^H-NMR (CDCl_3_): δ 7.37–7.19 (m, 11H), 6.96 (m, 1H), 6.35–6.33 (t, *J* = 7.2 Hz, 1H), 6.25–6.23 (d, *J* = 5.0 Hz, 1H), 5.08 (s, 1H), 4.40–4.38 (d, *J* = 5 Hz, 2H), 3.10 (s, 2H); ^13^C-NMR (CDCl_3_): δ 168.3, 151.2, 142.5, 140.4, 128.9, 128.5, 127.8, 110.7, 107.8, 55.0, 36.9, 36.2; LC-MS (ESI+) *m/z* 360 - [M+Na].

*2-Benzyhydrylsulfinyl-N-(furan-2-ylmethyl)acetamide* (**12a**). White liquid. R*_f_* = 0.08 (ethyl acetate/*n*-hexane = 2:1, v/v); IR _max_ (CHCl_3_, KBr): 2961, 2926, 1736, 1494, 1451, 1282, 1117, 1053, 1031, 967, 753 cm^−1^; ^1^H-NMR (CDCl_3_): δ 7.50–7.34 (m, 10H), 7.09 (s, 1H), 5.21 (s, 1H), 3.46–3.40 (d, *J* = 4.6 Hz, 1H), 3.18–3.12 (d, *J* = 4.6 Hz, 1H), 2.80–2.78 (d, *J* = 3.5 Hz, 3H); ^13^C-NMR (CDCl_3_): δ 164.9, 135.0, 129.7, 129.6, 129.0, 128.8, 72.3, 52.7, 26.6; LC-MS (ESI+) *m/z* 310 - [M+Na].

*2-Benzyhydrylsulfanyl-N-decylacetamide* (**12b**). White solid. mp 90.1 °C; R*_f_* = 0.4 (ethyl acetate/*n*-hexane = 1:1, v/v); IR _max_ (CHCl_3_, KBr): 3027, 2960, 2925, 1733, 1493, 1451, 1269, 1137, 1051, 1029, 750 cm^−1^; ^1^H-NMR (CDCl_3_): 7.50–7.31 (m, 10H), 7.07 (s, 1H), 5.19 (s, 1H), 3.45–3.39 (d, *J* = 4.9 Hz, 1H), 3.31–3.22 (q, *J* = 4.3 Hz, 2H), 3.15–3.09 (d, *J* = 4.3 Hz, 1H), 1.26 (brs, 16H), 0.90–0.85 (t, *J* = 3.5 Hz, 3H); ^13^C-NMR (CDCl_3_): δ 164.2, 134.9, 134.3, 129.7, 129.1, 128.8, 71.6, 52.5, 40.1, 32.1, 31.1, 29.7, 29.6, 27.1, 20.6, 14.3; LC-MS (ESI+) *m/z* 436 - [M+Na].

*2-Benzyhydrylsulfinyl-N-tetradecylacetamide* (**12c**). White solid. mp 93.1 °C; R*_f_* = 0.43 (ethyl acetate/*n*-hexane = 1:1, v/v); IR _max_ (CHCl_3_, KBr): 3298, 3061, 3027, 2127, 1951, 1736, 1599, 1494, 1450, 1368, 1274, 1124, 1027, 998, 752 cm^−1^; ^1^H-NMR (CDCl_3_): δ 7.51–7.33 (m, 10H), 7.08 (s, 1H), 5.20 (s, 1H), 3.45-3.40 (d, *J* = 4.8 Hz, 1H), 3.30–3.21 (q, *J* = 3.6 Hz, 2H), 3.15–3.09 (d, *J* = 3.6 Hz, 1H), 1.25 (brs, 24H), 0.90–0.85 (t, *J* = 3.2 Hz, 3H); ^13^C-NMR (CDCl_3_): δ 164.1, 135.0, 134.2, 130.8, 129.7, 128.9, 71.6, 52.6, 40.1, 32.1, 31.1, 29.7, 29.5, 27.1, 22.9, 14.4; LC-MS (ESI+) *m/z* 492 - [M+Na].

*N-Allyl-2-benzyhydrylsulfinylacetamide* (**12d**). Light yellow liquid. R*_f_* = 0.17 (ethyl acetate/*n*-hexane = 1:1, v/v); IR _max_ (CHCl_3_, KBr): 3493, 3285, 3063, 2924, 1662, 1563, 1495, 1451, 1413, 1316, 1161, 1031, 751 cm^−1^; ^1^H-NMR (CDCl_3_): δ 7.50–7.31 (m, 10H), 7.19 (s, 1H), 5.90–5.75 (m, 1H), 5.28–5.11 (m, 3H), 3.91–3.87 (t, *J* = 5.1 Hz, 2H), 3.48–3.43 (d, *J* = 3.9 Hz, 1H), 3.19–3.14 (d, *J* = 3.9 Hz, 1H); ^13^C-NMR (CDCl_3_): δ 164.2, 134.9, 134.2, 133.6, 129.7, 129.6, 129.1, 116.9, 52.7, 42.3, 31.1; LC-MS (ESI+) *m/z* 336 - [M+Na].

*2-Benzyhydrylsulfinyl-N-cyclohexylacetamide* (**12e**). White solid. mp 96.5 °C; R*_f_* = 0.27 (ethyl acetate/*n*-hexane = 1:1, v/v); IR _max_ (CHCl_3_, KBr): 3271, 3062, 2929, 2854, 2242, 1651, 1549, 1495, 1451, 1384, 1324, 1260, 1152, 1126, 1041, 985, 909, 891, 732 cm^−1^; ^1^H-NMR (CDCl_3_): δ 7.51–7.31 (m, 10H), 7.09 (s, 1H), 5.22 (s, 1H), 3.86–3.79 (m, 1H), 3.44–3.39 (d, *J* = 3.8 Hz, 1H), 3.13–3.08 (d, *J* = 4.2 Hz, 1H), 1.70–1.56 (q, *J* = 3.5 Hz, 4H), 1.40–1.13 (m, 6H); ^13^C-NMR (CDCl_3_): δ 163.1, 134.9, 129.7, 129.4, 128.6, 71.3, 52.4, 48.8, 32.7, 29.8, 24.8; LC-MS (ESI+) *m/z* 378 - [M+Na].

*2-Benzyhydrylsulfinyl-N-(tetrahydrofuran-2-ylmethyl)acetamide* (**12f**). White liquid. R*_f_* = 0.1 (ethyl acetate/*n*-hexane = 2:1, v/v); IR _max_ (CHCl_3_, KBr): 3287, 3064, 2926, 2871, 2243, 1738, 1667, 1556, 1495, 1451, 1383, 1304, 1257, 1136, 1080, 1031, 911, 732 cm^−1^; ^1^H-NMR (CDCl_3_): δ 7.53–7.27 (m, 11H), 5.28 (s, 1H), 4.03–3.99 (m, 1H), 3.88–3.82 (q, *J* = 3.8 Hz, 1H), 3.78–3.73 (q, *J* = 3.5 Hz, 1H), 3.49–3.42 (m, 2H), 3.19–3.10 (m, 2H), 2.04–1.86 (m, 4H); ^13^C-NMR (CDCl_3_): δ 164.5, 135.2, 129.4, 128.9, 128.7, 71.3, 68.2, 52.9, 52.6, 43.6, 28.8, 25.9; LC-MS (ESI+) *m/z* 380 - [M+Na].

*2-Benzyhydrylsulfinyl-N-[3-(2-oxo-pyrrolidin-1-yl)-propyl]acetamide* (**12g**). White liquid. R*_f_* = 0.2 (MC/MeOH = 10:1, v/v); IR _max_ (CHCl_3_, KBr): 3448, 3063, 2927, 1662, 1560, 1495, 1450, 1294, 1031, 751 cm^−1^; ^1^H-NMR (CDCl_3_): δ 7.52–7.21 (m, 11H), 5.28 (s, 1H), 3.44–3.19 (m, 8H), 2.41–2.35 (t, *J* = 3.5 Hz, 2H), 2.07–1.98 (m, 2H), 1.75–1.67 (m, 2H); ^13^C-NMR (CDCl_3_): δ 175.8, 164.2, 135.3, 129.4, 128.9, 128.6, 71.5, 54.1, 47.4, 39.9, 36.7, 31.0, 26.7, 18.0; LC-MS (ESI+) *m/z* 421 - [M+Na].

*2-Benzyhydrylsulfinyl-N-phenylacetamide* (**12h**). White solid. mp 100.7 °C; R*_f_* = 0.1 (ethyl acetate/*n*-hexane = 1:2, v/v); IR _max_ (CHCl_3_, KBr): 2922, 2851, 1736, 1676, 1601, 1552, 1498, 1445, 1384, 1327, 1247, 1123, 1029, 906, 755 cm^−1^; ^1^H-NMR (CDCl_3_): δ 9.14 (s, 1H), 7.56–7.11 (m, 15H), 5.22 (s, 1H), 3.69–3.63 (d, *J* = 3.6 Hz, 1H), 3.28–3.23 (d, *J* = 3.6 Hz, 1H); ^13^C-NMR (CDCl_3_): δ 162.3, 155.7, 141.7, 133.8, 131.2, 129.2, 128.7, 124.9, 120.4, 67.7, 44.1; LC-MS (ESI+) *m/z* 372 - [M+Na].

*2-Benzyhydrylsulfinyl-N-(4-ethylphenyl)acetamide* (**12i**). White solid. mp 168.1 °C; R*_f_* = 0.38 (ethyl acetate/*n*-hexane = 1:1, v/v); IR _max_ (CHCl_3_, KBr): 3259, 3190, 3121, 3062, 2962, 2924, 2852, 1675, 1608, 1544, 1514, 1451, 1413, 1325, 1119, 1032, 908, 834 cm^−1^; ^1^H-NMR (CDCl_3_): δ 7.71–7.35 (m, 12H), 7.26 (s, 1H), 7.15–7.12 (d, *J* = 7.5 Hz, 2H), 5.26 (s, 1H), 3.68–3.62 (s, 1H), 3.26–3.20 (d, *J* = 4.5 Hz, 1H), 2.67–2.57 (q, *J* = 3.7 Hz, 2H), 1.22 (t, *J* = 3.3 Hz, 3H); ^13^C-NMR (CDCl_3_): δ 162.3, 141.0, 135.9, 134.1, 129.7, 129.2, 129.0, 128.6, 120.6, 71.8, 51.7, 28.6, 15.9; LC-MS (ESI+) *m/z* 400 - [M+Na].

*2-Benzyhydrylsulfinyl-N-(4-dimethylaminophenyl)acetamide* (**12j**). Yellow solid. mp 184.3 °C; R*_f_* = 0.17 (ethyl acetate/*n*-hexane = 1:1, v/v); IR _max_ (CHCl_3_, KBr): 3288, 3063, 2922, 1666, 1603, 1519, 1450, 1326, 1223, 1163, 1124, 1031, 946, 818, 749 cm^−1^; ^1^H-NMR (CDCl_3_): δ 9.03 (s, 1H), 7.52–7.33 (m, 12H), 6.68–6.64 (d, *J* = 7.1 Hz, 2H), 5.31 (s, 1H), 3.64–3.59 (d, *J* = 3.4 Hz, 1H), 3.38–3.29 (d, *J* = 3.4 Hz, 1H), 2.91 (s, 6H); ^13^C-NMR (CDCl_3_): δ 161.8, 148.0, 134.8, 129.8, 129.5, 129.0, 128.9, 121.8, 113.1, 71.3, 52.7, 41.1; LC-MS (ESI+) *m/z* 415 - [M+Na].

*2-Benzyhydrylsulfinyl-N-(furan-2-ylmethyl)-acetamide* (**12k**). Yellow liquid. R*_f_* = 0.2 (ethyl acetate/*n*-hexane = 1:1, v/v); IR _max_ (CHCl_3_, KBr): 3298, 3062, 3030, 2927, 2856, 1730, 1660, 1554, 1539, 1495, 1452, 1384, 1315, 1030, 749 cm^−1^; ^1^H-NMR (CDCl_3_): δ 7.53 (s, 1H), 7.41–7.26 (m, 11H), 6.34–6.32 (t, *J* = 6.8 Hz, 1H), 6.28–6.27 (d, *J* = 6.8 Hz, 1H), 5.16 (s, 1H), 4.65–4.57 (q, *J* = 4.5 Hz, 1H), 4.38–4.30 (q, *J* = 4.1 Hz, 1H), 3.52–3.46 (d, *J* = 3.8 Hz, 1H), 3.12–3.06 (d, *J* = 3.8 Hz, 1H); ^13^C-NMR (CDCl_3_): δ 164.3, 152.8, 142.4, 134.7, 130.0, 129.0, 128.8, 110.7, 108.0, 71.3, 51.8, 36.7; LC-MS (ESI+) *m/z* 376 - [M+Na].

### 3.3. Biology

#### 3.3.1. BV-2 Microglia Culture

The murine BV-2 microglia cell line was maintained in DMEM supplemented with 10% FBS and penicillin/streptomycin at 37 °C in a humidified incubator under 5% CO_2_. For all experiments, cells were plated at a density of 1 × 10^5^ cells/mL in 24-well plates and then treated with 100 ng/mL LPS alone or with various concentrations of compounds for 24 h at 37 °C.

#### 3.3.2. Nitrite Assay

The Griess reaction was used to perform nitrite assays as indication of nitrite accumulation in the medium. BV2 cells were incubated with LPS (lipopolysaccharide, 100 ng/mL) and various concentrations of modafinil derivatives and aspirin for 24 h at 37 °C. After induction of inflammation by LPS in cells, the levels of generated nitrite would be elevated. The culture media was then mixed with an equal volume of reagent (1 part 0.1% *N*-1-naphthylethylenediamine dihydrochloride, 1 part 1% sulfanilamide in 5% phosphoric acid) in 96-well plates. The absorbance was determined at 540 nm using a microplate reader. Data are reported as the mean ± the standard deviation of three observations.

#### 3.3.3. Polymerase Chain Reaction for iNOS and COX-2

BV2 cells were stimulated with LPS in the absence or presence of modafinil derivatives for 24 h. Total RNA was isolated from cells using TRIzol (Invitrogen, Carlsbad, CA, USA) according to the manufacturer’s instructions. For cDNA synthesis, 2 µg of total RNA was reverse-transcribed using the SuperScript First-Strand Synthesis System (Invitrogen). cDNA was amplified by polymerase chain reaction (PCR) using primers for iNOS and COX-2; cDNA was amplified by polymerase chain reaction (PCR) using primers for iNOS (F: GTGTTCCACCAGGAGATGTTG, R: CTCCT GCCCACTGAGTTCGTC) and COX-2 (F: AAGACTTGCCAGGCTGAACT, R: CTT CTGC AGTCCAGGTTCAA). PCR products were separated by 1% agarose gel electrophoresis and visualized by ethidium bromide staining.

## 4. Conclusions

In conclusion, we have demonstrated a new and practical synthetic route to modafinil (**2**) using readily available inexpensive reagents and simple reaction conditions that do not require any special equipment or techniques. We expect that this method will prove to be useful for the practical preparation of modafinil and modification of its derivatives. We have found that the modafinil derivatives **11a–k** and **12a–k** exhibited anti-inflammatory evidenced by lowering of LPS-stimulated nitrite production in BV2 microglial cells. The anti-inflammatory activity of modafinil derivatives is better than that of aspirin in these cultured cells. Interestingly, the sulfoxide analogues **12a–k** showed more favorable activity than the sulfide analogues **11a–k**. In case of sulfide analogues, introduction of an aliphatic group on the amide part in **11a–d** resulted in lower anti-inflammatory activity compared with cyclic or aromatic moieties (compounds **11e–k**). However, for the sulfoxide analogues, introduction of aliphatic moieties (compounds **12a–d**) resulted in higher anti-inflammatory activity than cyclic or aromatic fragments (compounds **12e–k**).
